# Assessing home-based rehabilitation within the development of an integrated model of care for people living with HIV in a resource-poor community

**DOI:** 10.4102/phcfm.v9i1.1374

**Published:** 2017-08-31

**Authors:** Saul Cobbing, Jill Hanass-Hancock, Hellen Myezwa

**Affiliations:** 1 Department of Physiotherapy, Westville Campus, University of KwaZulu-Natal, South Africa; 2 HIV Prevention Research Unit, Medical Research Council of South Africa, South Africa; 3 Department of Physiotherapy, University of the Witwatersrand, South Africa

## Abstract

**Background:**

People living with HIV (PLHIV) are living longer lives but are at a greater risk of developing disability. South Africa has the largest antiretroviral therapy (ART) programme in the world, shifting HIV from a deadly to a chronic disease. The integration of rehabilitation into chronic care is therefore now crucial to ensure the highest quality of life of PLHIV.

**Aim:**

To describe how a home-based rehabilitation (HBR) programme adhered to the fundamental principles of a theoretical model of integrated care developed for the study setting in KwaZulu-Natal, South Africa.

**Method:**

The process and results from the HBR programme were assessed in relation to the model of care to ascertain which principles of the model were addressed with the HBR programme and which elements require further investigation.

**Results:**

The HBR programme was able to apply a number of principles such as evidence-based practice, task shifting to lay personnel, enabling patient-centred care and maximising function and independence of PLHIV. Other elements such as the adoption of a multidisciplinary approach, training on the use of disability screening tools and the use of evidence to influence policy development were more difficult to implement.

**Conclusion:**

It is possible to implement elements of the integrated model of care. Further research is needed to understand how principles that require further training and collaboration with other stakeholders can be implemented. The results of this study provide additional evidence towards understanding the feasibility of the theoretical model and what is required to adjust and test the full model.

## Background

Following more than a decade of activism by human rights’ and civil society organisations,^1^ 3.4 million people living with HIV (PLHIV) in South Africa have thus far gained access to the largest antiretroviral therapy (ART) programme in the world. HIV has shifted from a deadly disease to a chronic condition requiring lifelong medication. The population currently on ART constitutes approximately half of the total number of PLHIV in South Africa.^2^ Even greater access to ART could be achieved by the recent removal of CD4 criteria from South African ART treatment guidelines,^3^ which further re-emphasises the approach of universal access to ART. This policy is also promoted thorough the ambitious UNAIDS 90-90-90 targets^4^ and the new World Health Organization (WHO) guidelines on antiretroviral treatment.^5^ However, activists, healthcare workers (HCWs) and scientists in South Africa are now shifting their attention beyond access to treatment towards increased access to a continuum of care that ensures quality of life for PLHIV.^6^ Lazarus et al.^7^ propose a ‘fourth 90’ target, which should aim to ensure that 90% of PLHIV with viral suppression have good health-related quality of life, while Hanass-Hancock et al.^8^ call for 100% of services for PLHIV to be able to link to mitigating services like rehabilitation.

Enhancing the quality of life of PLHIV requires, among other interventions, the integration of rehabilitation in the continuum of care, an aspect of care that has often been neglected in the emergency-type of response to HIV. Improved access to ART has resulted in PLHIV living longer lives, but this comes with new health challenges related to chronicity^9,10^ and an increased risk of developing physical and cognitive disabilities.^11^ Recent WHO guidelines^5^ recognise this development, calling for the integration of rehabilitation into the continuum of care for PLHIV. In South Africa, Goal 1 of the Framework and Strategy on Disability and Rehabilitation Services^12^ explicitly mentions integrating rehabilitation and disability services into priority health programmes, which include HIV care. This framework is supported by the new National Strategic Plan for STIs, HIV and TB,^13^ which focuses on the reduction of disability resulting from HIV and TB (objective 3). The proposed National Health Insurance^14^ further promotes the rights of all South Africans to access quality healthcare services that ‘are affordable without exposing them to financial hardships’. Healthcare providers working in South Africa are also compelled, by the recently published White Paper on the Rights of Persons with Disabilities,^15^ to ensure that all HIV programmes are accessible to persons with disabilities. These documents all promote the integration of accessible disability and rehabilitation services into the HIV care. They do not describe, however, how these services can be integrated nor which rehabilitation services or approaches may be feasible to address some of the barriers that PLHIV face in accessing holistic healthcare. The challenge therefore has emerged to understand what kind of model of care can link chronic care and rehabilitation and which rehabilitative approaches are effective and feasible for such a model of care in resource-poor settings.

In response to this challenge, a team of South African-based researchers at the University of KwaZulu-Natal and the University of the Witwatersrand developed a larger research project that attempted to describe the needs of rehabilitation for PLHIV and understand which model of care would best integrate chronic HIV and rehabilitation services. The project (with all its sub-studies) was located in a semi-rural, resource-poor area of KwaZulu-Natal province, South Africa. The larger project included an exploratory study,^16^ a longitudinal cohort-study,^11,17^ the development of a theoretical model of integrated care^18,19^ and a training approach for HCWs.^20^ The development of a theoretical model of care included a thorough review of international rehabilitation policies and consensus with a wide range of key local stakeholders and experts.^18^ This will henceforth be referred to in this article as the Chetty model. The need for this new model was driven by the fact that a large number of PLHIV in this area experience functional limitations,^11^ while they were experiencing obstacles in accessing institutional-based rehabilitation, including physical challenges, high costs and accessibility of healthcare and supporting services.^16^ No previous research has proposed a model of care that addresses the service delivery for rehabilitation among people with HIV and their unique needs. The authors concede that there are many aspects of service delivery that are common to HIV and other chronic diseases, but specific factors related to HIV such as the high prevalence, stigma and existing programme approaches demand that service providers and researchers address the unique needs of this patient population.

**FIGURE 1:**
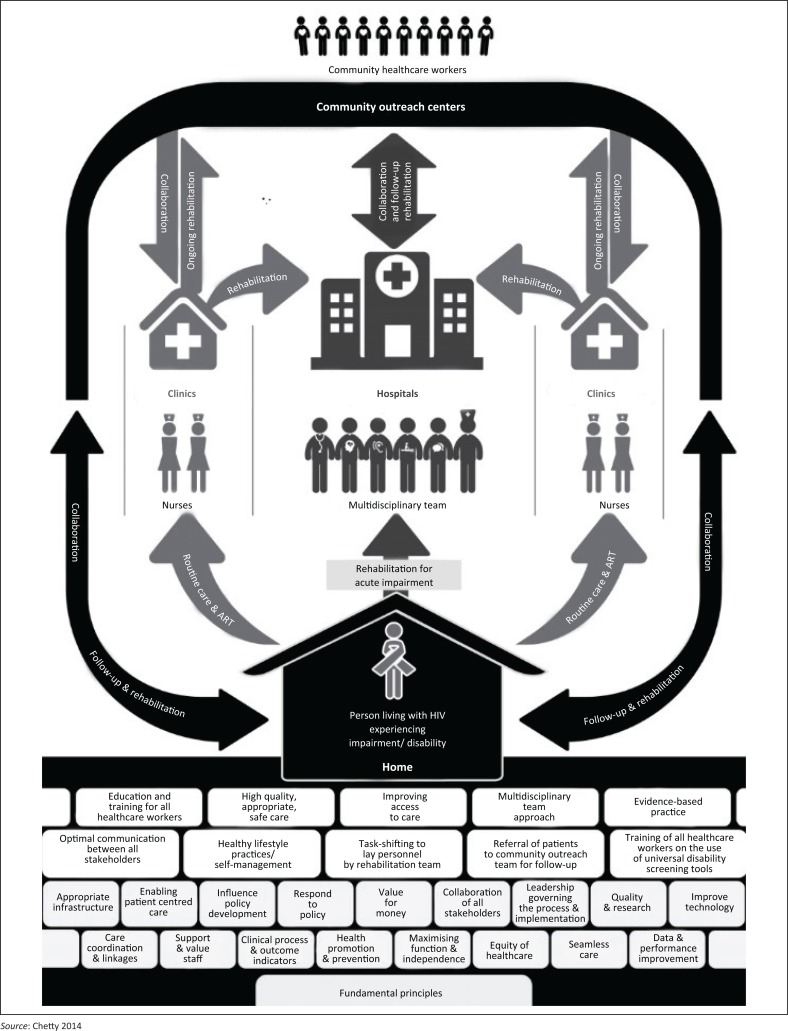
Model of care for the rehabilitation of PLHIV in a semi-rural South African setting (Chetty model). Reproduced with permission from the author.^18^

The Chetty model promotes greater integration and collaboration between hospitals and communities. It also supports alternative approaches such as task shifting, a strategy that aims to train community care workers (CCWs) to deliver varied health services to PLHIV experiencing disability in resource-poor settings. The Chetty model was a result of a series of consultative and interlinked research projects that aimed to understand living long-term with HIV in a semi-rural setting in KwaZulu-Natal, South Africa. The studies that were located at the project setting and contributed to the development of the Chetty model are summarised in Table 1. Figure 1 depicts the Chetty model. A number of studies^11,16,20,21,22^ contributed to the development of this model, which further evolved through systematic design^23^ and review^24^ processes. The rehabilitation of PLHIV in their own homes is seen as central to the implementation of the model. The aim of this article is to describe how a home-based rehabilitation (HBR) programme designed for PLHIV in this study community adhered to the fundamental principles of the Chetty model. This HBR intervention was the first attempt to test part of this model in practice.

**TABLE 1:** Studies contributing to the development of the Chetty model.

Author (year)	Objective	Method	Participants	Key outcomes
Hanass-Hancock et al. (2012)^21^	To understand living long-term with HIV while accessing ART using a disability lens (Nathi Singabantu study)	Qualitative (semi-structured interviews)	Adult PLHIV on ART	PLHIV experience a diverse set of functional limitations that may need rehabilitation
Cobbing et al. (2014)^16^	To describe the experiences of PLHIV who received hospital-based physiotherapy with the aim of informing future rehabilitation interventions	Qualitative (semi-structured interviews)	Adult PLHIV	Hospital-based physiotherapy rehabilitation is hampered by a number of barriers to patients
Hanass-Hancock et al. (2014)^20^	To sensitise HCWs and people with disabilities on the inter-relationship of HIV and disability	Quantitative (short checklist) and qualitative (semi-structured interviews)	HCWs and people with disabilities in study hospital and surrounding community	Training presented opportunities to improve services for people with disabilities and also provided knowledge and skills to initiate improvements
Van Egeraat et al. (2015)^22^	To understand the perspective of HCWs with regard to HIV-related disabilities	Qualitative (semi-structured interviews	Variety of healthcare professionals in the study area (nurses, rehabilitation staff, managers and community healthcare)	All HCWs identified HIV-related disabilities as an issue in their daily work with PLHIV. The lack of training, skills and resources for providing rehabilitation services was identified
Hanass-Hancock et al. (2015)^11^	To understand the scope of functional limitations experienced by PLHIV on ART	Cross-sectional survey and longitudinal cohort study	Adult PLHIV on ART (from four local clinics in study community)	35% of PLHIV on ART experienced some type of functional limitations; these are associated with key outcomes of livelihood, ART adherence, health and depressive symptoms. Access to rehabilitation services is lacking in the area
Chetty et al. (2014)^23^	To develop a model of care for the rehabilitation of PLHIV in a semi-rural African setting	Mixed methods using a Learning in Action Approach and Delphi technique	Local HIV experts, multidisciplinary healthcare team, department of health representatives; non-governmental organisation representative and service users at the study setting	Design article (no outcomes)
Chetty et al. (2015)^24^	To provide an overview of current models of rehabilitative care and examine how these can inform the inclusion of rehabilitation into a model of care for PLHIV within a public healthcare South African framework	Review article	None	The need to develop a model to guide rehabilitation of PLHIV in South Africa, involving multiple stakeholders, is essential to address the cumulative disabling effects of the virus and its treatment.

ART, antiretroviral therapy; HCWs, healthcare workers; PLHIV, people living with HIV.

### Sources of evidence informing the scope of this article

This theoretical article is the fifth and concluding article in a series of articles contributing to a doctoral study focusing on HBR for PLHIV. The study area is situated in the same resource-poor community in which the Chetty model was developed. The study aimed to design an HBR intervention for adult PLHIV as a pathway to comprehensive care and to determine the effect of this intervention on PLHIVs’ perceived disability, quality of life, functional mobility and capacity. The study process included four distinct steps: a scoping review of available literature,^25^ a description of the study methodology^26^ and the presentation of both quantitative and qualitative study results.^27,28^ Table 2 summarises the four articles that were undertaken as part of the overarching doctoral study. This final article assesses to what extent the study was able to implement the principles of the Chetty model.^18^

**TABLE 2:** Summary of study articles.

Author (year)	Objective	Method	Participants	Key outcomes
Cobbing et al. (2016)^25^	To summarise the evidence related to the effectiveness of HBR interventions designed specifically for adult PLHIV	Scoping review method	None (scoping review)	A small number of articles (six) suggest that HBR is a safe management option that may confer a number of physical and psychological benefits for adult PLHIV
Cobbing et al. (2015)^26^	To describe the design of a novel HBR intervention for adult PLHIV in a resource-poor South African setting	Design article	None (design article)	Design article (no outcomes)
Cobbing et al. (2016)^27^	To investigate the effects of a 16-week HBR intervention on the quality of life, functional mobility and functional capacity of adult PLHIV in KwaZulu-Natal, South Africa	Single-blinded randomised controlled trial design	Adult PLHIV with mobility limitations	HBR for PLHIV is a safe means of addressing the functional deficits experienced by PLHIV and appears likely to improve quality of life
Cobbing et al. (2017)^28^	To explore CCWs’ experiences of being involved in carrying out a HBR intervention for PLHIV	Qualitative (semi-structured interviews)	CCWs	Participants described the factors that enabled them to successfully implement the intervention as well as a number of inhibitors

CCWs:, community care workers; HBR, home-based rehabilitation; PLHIV, people living with HIV.

The systematic scoping of the existing literature revealed a paucity of evidence related to HBR interventions for PLHIV^25^ with only six articles meeting the review’s inclusion criteria. Synthesis of the evidence from this review and the practical experience of the lead author as a rehabilitation practitioner working with PLHIV informed the design of the study intervention, outlined in detail in the methodological article.^26^ This design ensured that the study intervention would be of a high methodological quality (as required by the Chetty model), scoring 8 out of a possible 10 points on the PEDro scale, a tool employed to assess the quality of randomised controlled trials (RCTs) (Maher 2003). The intervention study itself used an RCT design assigning people to either an HBR group or a group that received information material. The results indicate that HBR is a safe strategy for treating the functional limitations experienced by PLHIV and may further improve their quality of life.^27^ Collected data also showed through within-group changes that the group receiving HBR (intervention group) improved more than the group receiving health information (control group) across all outcome measures. These results demonstrate the clinical significance of this HBR intervention for adult PLHIV, which can be defined as evidence that an intervention has a beneficial impact on individuals exposed to it, thus representing the ability to make a difference in their lives.^29^ Statistically, however, between-group differences were non-significant. Non-significance of results may have been because of the trial time (16-week intervention) not being long enough or the fact that participants in the intervention and control groups came from the same community, which may have led to communication between groups. Furthermore, for ethical reasons, participants in the control group received health information on exercise and lifestyle, which may have explained the improvements in this group.

A task shifting approach was employed, in which four CCWs who lived in the study community received training from a qualified physiotherapist to enable them to implement this HBR programme (training is also a requirement of the Chetty model). This training, conducted over a four-week period, included theoretical instruction on research ethics, basic anatomy and HIV-related pathology, as well as practical skills including strength and aerobic exercise prescription. The experiences of the CCW involved in this programme are further described in a qualitative article.^28^ Participants reported feeling empowered by the knowledge and skills they attained through this study and gave valuable advice for improving future interventions in this field.

## Methods

For the purposes of this article, the authors use the Chetty model to assess if the intervention was able to apply the fundamental principles of the model. A comparison of the overall study in relation to the Chetty model was undertaken using a desk review to assess how the process and results of the study adhere to the fundamental principles of this model as well as to highlight areas that were not adequately addressed. The first author took the lead on this task, after which the two co-authors (who were research supervisors for this study and who have conducted other research in this area) analysed and revised this comparison where necessary.

## Findings

Table 3 indicates which of the principles of the Chetty model this study was able to implement and how each principle was addressed.

**TABLE 3:** Principles of Chetty model addressed by study.

Principle	Implemented	Description	Not fully implemented	Description
**Education training for all HCWs**	✔	CCWs trained before and during intervention^26^	-	-
**High quality, appropriate, safe care**	✔	RCT shown to be safe and beneficial to intervention group^26^	-	-
**Improving access to care**	✔	Participants received rehabilitation in their own homes^27^	-	-
**Multi disciplinary team approach**		-	✔	Training or supervision of CCWs primarily by physiotherapist
**Evidence-based practice**	✔	Rigorous study design based on strong evidence base^25,26^	-	-
**Optimal communication between stakeholders**	✔	All stakeholders at research site informed of study progress before, during and after study^27^	-	-
**Healthy lifestyle practices or self-management**	✔	Participants taught self-management of health^27^	-	-
**Task shifting to lay personnel**	✔	A central tenet of this study was the training of CCWs to implement intervention^26,27^	-	-
**Referral of patients to community outreach**	✔	Participants referred to outreach (provided by the local NGO) when necessary^27^	-	-
**Training of HCWs on use of disability screening tools**	-	-	✔	Participants screened for disability in previous cohort
**Appropriate infrastructure**	✔	Safe, appropriate venue for pre- and post-intervention testing. Equipment provided when necessary to participants^27^	-	-
**Enabling patient-centred care**	✔	Close communication with participants and the provision of needs-based rehabilitation^27^	-	-
**Influencing policy development**	-	-	✔	This is yet to be fully achieved; however, the study results have been used to inform the new South African NSP for HIV^13^
**Respond to policy**	✔	CBR is central to both national and global policies^25^		
**Value for money**	-	-	✔	The cost-effectiveness of this intervention is being analysed (results are not available at present)
**Collaboration of all stakeholders**	✔	All stakeholders at the research site were consulted about the overall study design^26^		
**Leadership governing process of implementation**	-	-	✔	This is yet to be fully achieved. Broader implementation will be sought after further research has been conducted and policymakers have been engaged
**Quality and research**	✔	High-quality RCT (score of 8 out of 10 on PEDro scale) conducted and complemented by additional qualitative research methods^27,28^		
**Improve technology**	-	-	✔	Communication was not optimal between CCWs and HBR participants, resulting in missed appointments and participants not attending testing
**Care coordination and linkages**	✔	Participants referred to both local NGO and district hospital for further care when required^27^	-	-
**Support and value staff**	✔	CCWs reported personal growth from their involvement in the study^28^	-	-
**Clinical process and outcome indicators**	✔	Valid and reliable outcome measures were employed^26^	-	-
**Health promotion and prevention**	✔	Participants in both groups received information on healthy living^26, 27^	-	-
**Maximising function and independence**	✔	This was the main focus of the specific rehabilitation provided to each participant^27^	-	-
**Equity of healthcare**	✔	Participants received high-quality free care in their own homes^27^	-	-
**Seamless care**	✔	Participants referred for further care when required^27^	-	-
**Data and performance improvement**	✔	Participant diaries utilised and extensive data collected on outcome measures pre- and post-intervention, as well as interviews of CCWs^26, 27, 28^	-	-

CBR, community-based rehabilitation; CCWs, community care workers; HBR, home-based rehabilitation; HCWs, healthcare workers; NGO, non-governmental organisation; NSP, National Strategic Plan; PEDro, Physiotherapy Evidence Database; RCT, randomised controlled trial.

The elements of the Chetty model that were implemented in this study included the training of HCWs, task shifting to lay workers, support of staff, improved access to safe patient-centred care, linkages to institutional care and community outreach, as well as a specific focus on maximising the function and independence of study participants. The publications from this study further reveal that the HBR programme was based on a sound evidence-based information^25^ and responded to relevant local and international policies related to community-based rehabilitation,^30^ primary healthcare,^14,31^ task shifting^32^ and HIV management.^5,12^ The study process included a high-quality, rigorous RCT research method, with the data obtained from valid and reliable outcome indicators. It is also important to acknowledge that the study benefited from being part of the overall project and was conducted in a setting where the researchers had worked for a number of years, where healthcare staff had been trained on the relationship between HIV and disability and were thus more receptive to alternative rehabilitation solutions. This fact may also have limited the objectivity of the researchers conducting this appraisal.

## Discussion

The assessment described above suggests that the development and implementation of the HBR programme were able to adhere to the majority of the fundamental principles of the Chetty model. The fact that trained CCWs were able to safely implement the HBR intervention provides empirical support for a task shifting approach to the rehabilitation management of PLHIV. Task shifting has been defined as the reassignment of specific tasks to different cadres of HCWs^33^ and has been proposed as a solution to the shortage of health professionals in South Africa.^34^ Indeed, it has been demonstrated in the same study location that appropriately trained lay counsellors can effectively deliver group-based counselling for PLHIV with co-morbid depression.^35^ Similarly the Framework and Strategy on Disability and Rehabilitation Services in South Africa^12^ promotes the rehabilitation of patients in their own homes and the training of CCWs to detect disability and refer appropriately. The participants recruited for the HBR intervention in this study were selected from an existing cohort who had already been screened for potential mobility limitation.^11^ The CCWs working on the intervention were not trained to identify disability, however, and thus the study did not include a fundamental principle of the Chetty model of care, namely to train all workers on the use of disability screening tools. Hence, future research needs to identify feasible strategies using simplified screening and referral tools to enable and train healthcare staff at all levels to screen, identify and refer individuals with functional limitations. The pilot workshops included in the overall project echo this analysis as they revealed that HCWs can address certain elements of disability-inclusive healthcare services (for example the creation of disability help desks) but that others elements of care such as screening for disability and referral to rehabilitation services needs more long-term planning and innovation.^20^

Another key principle of the Chetty model of care that could not be implemented in this study was the full adoption of a multidisciplinary team approach in the design and implementation of the HBR intervention. While the pre- and post-intervention testing for the RCT assessed a broad range of outcome measures, assessing physical, spiritual and psychological factors, the intervention focused primarily on providing rehabilitation that addressed participants’ physical and functional limitations, reflecting the background of the lead author, a qualified physiotherapist. The lead author also functioned as the supervising rehabilitation specialist, who conducted the training of the CCWs and supervised them throughout the 16-week programme. In order to better reflect a holistic and integrated approach of the Chetty model, the research team needs to develop innovations that will involve collaboration with the full cross-section of health professionals. Integration could for instance be facilitated through enhanced training, which allows CCWs to employ simple screening tests that assess an individual’s physical function, psychological state and social needs. A trained CCW could also provide counselling and facilitate referral to an appropriate professional. This screening could take place during routine clinic visits, at patients’ homes or even in patient adherence clubs, where both screening and rehabilitation services could be provided.

The Chetty model of care also recommends that alternative rehabilitation interventions for PLHIV should represent good value for money for state and external funders. With a specific focus on HIV care in Africa, two separate reviews^33,36^ concluded that task shifting will ensure cost-effectiveness and sustainability of interventions, without compromising patient care. Indeed, studies of HBR interventions for other chronic disease populations^37,38,39^ have shown that these interventions are cost-effective, if not more so, than traditional institution-based interventions. It is important, therefore, that research funds are allocated to measure the cost of novel rehabilitation interventions for PLHIV in comparison to current practice, in addition to assessing outcome measures of health, quality of life and function. A cost–benefit analysis of the HBR intervention implemented in this study is currently being conducted, but no results were available at the time of writing. Where possible, all rehabilitation interventions that provide an alternative to the standard of care should be assessed for cost-effectiveness, in order to enable policymakers and stakeholders to assess the feasibility of taking up these approaches in the public sector. The Chetty model needs to be supported by more practical approaches on how to collect this kind of data efficiently.

Implementing the Chetty model should take advantage of existing initiatives for integrated community care, and this is indeed a potential addition that could be made to the Chetty principles. For instance the South African Department of Health has recently trialled ward-based outreach teams, comprising six CCWs supervised by one nurse, to improve health promotion and disease prevention in resource-poor communities.^40^ While this initiative has shown some success, it has been argued that the role of the CCWs involved in this initiative is too narrow and their scope should be broadened to include curative functions.^41^ By including information on rehabilitation and disability assessment into the training of the CCW involved in these ward-based outreach teams, the rehabilitative options available to all people living with disability in under-resourced communities would be increased. The Chetty model could be implemented using such structures and, through doing this, benefit all people who have functional limitations or disabilities (not only those with HIV). This may potentially reduce the cost burden to the public health service in the longer term, by ensuring that these individuals do not develop more severe disabilities that incur high treatment costs.

Those principles that involved a larger component of stakeholders, access to technology or collaboration with policymakers were more difficult to implement within the doctoral study context. Some of those will be implemented, however, within the larger project, and therefore further follow-up and assessment of the implementation of the Chetty model over time in this context will be very beneficial to understand if and how this theoretical model can be implemented.

## Conclusion

This article has shown how the HBR intervention, designed and assessed in this study, complements a wider model of chronic care for PLHIV that has been developed in the same resource-poor location. Study participants were provided with safe, evidence-based and accessible patient-centred rehabilitation using a task shifting paradigm that empowered and trained lay care workers from the study community. The assessment of this study within the wider Chetty model has the potential to further inform pragmatic implementation strategies that may enhance the health-related quality of life of PLHIV and provide alternative rehabilitation options, particularly for people experiencing disability in resource-poor communities. Such a trial needs to integrate the approach from this study and other fundamental principles of the model of care that this HBR intervention did not seek to address. These elements include the adoption of a multidisciplinary approach throughout the research process, the investigation of programme cost-effectiveness and potential training approaches for disability screening. Evidence of this nature could then be used to inform policy development and ultimately the translation of theory into practice. As more and more PLHIV gain access to effective treatment and live longer lives, it is imperative that HCWs, advocates, researchers, communities and wider stakeholders form collaborations that focus on providing the appropriate policy and operational structures to ensure that rehabilitation is widely included in the management and care of this population. This will give PLHIV the best possible chance of achieving a better health-related quality of life and the opportunity to participate more fully in all of their life pursuits.
